# Effect of combined treatment with bisphosphonate and vitamin D on atherosclerosis in patients with systemic lupus erythematosus: a propensity score-based analysis

**DOI:** 10.1186/s13075-018-1589-9

**Published:** 2018-04-17

**Authors:** Kazumasa Ohmura, Masaru Kato, Toshiyuki Watanabe, Kenji Oku, Toshiyuki Bohgaki, Tetsuya Horita, Shinsuke Yasuda, Yoichi M. Ito, Norihiro Sato, Tatsuya Atsumi

**Affiliations:** 1 Department of Rheumatology, Endocrinology and Nephrology, Faculty of Medicine and Graduate School of Medicine, Hokkaido University, Sapporo, Japan; 2 Department of Biostatistics, Faculty of Medicine and Graduate School of Medicine, Hokkaido University, Sapporo, Japan; 3 Hokkaido University Hospital Clinical Research and Medical Innovation Center, Sapporo, Japan

**Keywords:** Systemic lupus erythematosus, Atherosclerosis, Osteoporosis, Bisphosphonates, Vitamin D

## Abstract

**Background:**

Premature atherosclerosis is one of the major complications of systemic lupus erythematosus (SLE). Recently, the biological linkage between atherosclerosis and osteoporosis has garnered much attention. The aim of this study is to explore correlation between the development of atherosclerosis and anti-osteoporotic treatment.

**Methods:**

Consecutive patients with SLE (n = 117) who underwent carotid ultrasonography were retrospectively analyzed using propensity scoring.

**Results:**

Of the 117 patients, 42 (36%), 27 (23%), and 30 (26%) were receiving bisphosphonates and vitamin D (BP + VD), bisphosphonates alone, or vitamin D alone, respectively. Low bone mineral density was more frequent, and carotid plaque was less prevalent in the BP + VD group compared with other treatment groups. Age (OR = 1.57) and BP + VD treatment (OR = 0.24) were shown by multivariate analysis to be associated with the presence of carotid plaque. In all strata divided using the propensity score, carotid plaque was statistically significantly less prevalent (*p* = 0.015, Mantel-Haenszel test) in the BP + VD group relative to the other treatment groups.

**Conclusion:**

Combined treatment with bisphosphonate and vitamin D may have a role in preventing atherosclerosis in patients with SLE.

**Electronic supplementary material:**

The online version of this article (10.1186/s13075-018-1589-9) contains supplementary material, which is available to authorized users.

## Background

Preventing atherosclerosis is a key objective while monitoring patients with systemic lupus erythematosus (SLE). In addition to traditional risk factors, such as hypertension and diabetes mellitus, SLE-related risk factors including glucocorticoid use, disease activity, antiphospholipid antibodies, and renal manifestations, have been shown to promote atherosclerosis [1]. Owing to progress in the treatment of SLE, cardiovascular disease (CVD) has replaced lupus-related organ failure to become the main cause of morbidity and mortality in these patients.

The biological link between atherosclerosis and osteoporosis has been reported previously [2, 3]. As with the β-hydroxy β-methylglutaryl-CoA (HMG-CoA) reductase inhibitors (“statins”), nitrogen-containing bisphosphonates have been known to inhibit the mevalonate pathway through interaction with farnesyl pyrophosphate synthase and are therefore expected to interfere with intimal plaque formation [4]. Vitamin D deficiency is emerging as a novel risk factor for CVD [5]. However, it remains controversial whether bisphosphonate (BP) therapy or vitamin D (VD) supplementation is prophylactic against atherosclerosis. Currently, there are no data to establish correlation between the development of atherosclerosis and treatment with BP and/or VD, which are frequently prescribed in patients with lupus. We hypothesized that combined treatment with BP + VD may be negatively associated with the progression of atherosclerosis in patients with SLE and herein report an unbiased study using propensity scoring.

## Methods

### Patients

Of the 220 consecutive patients with SLE who received glucocorticoids (GC) at the Hokkaido University Hospital between January 2013 and August 2015, 117 underwent carotid ultrasonography to assess subclinical atherosclerosis. All 117 patients met the 1997 American College of Rheumatology (ACR) revised criteria for SLE [6, 7].

### Study design

This study was designed as a cross-sectional study in a single center to examine whether BP + VD was protective against atherosclerosis compared with other treatments for osteoporosis in patients with SLE. This study was approved by the local ethical committee of Hokkaido University (approve number 015–0459). Clinical data were retrospectively reviewed at the time of undergoing carotid ultrasonography. Traditional risk factors for atherosclerosis were defined as the presence of hypertension, diabetes mellitus, dyslipidemia, smoking, increased body mass index, or history of CVD. SLE-related risk factors for atherosclerosis included duration of disease, duration of GC use, disease activity assessed by SLE disease active index 2000 (SLEDAI-2 K), presence of antiphospholipid antibodies, and renal manifestations, as previously reported [1].

### Carotid ultrasonography

Carotid plaque was defined as a localized lesion with maximum thickness of more than 1 mm, which had a point of inflection on the surface of the carotid intima-media complex. Measurement of mean carotid intima-media thickness (IMT) was performed on the bilateral common carotid arteries. The mean IMT was defined as the average of three points where plaque was absent [8].

### Bone mineral density measurements

Bone mineral density (BMD) was measured by dual-energy x-ray absorptiometry at the lumbar spine (L2–4 anteroposterior view) and/or femoral neck in accordance with standard instrument procedures. All BMD measurements were expressed as absolute values in gram of bone mineral per square centimeter (g/cm^2^) and as the T-score, the number of standard deviations (SD) above or below the mean value for young adult women. According to World Health Organization criteria, low BMD (defined as including osteopenia and osteoporosis), osteopenia and osteoporosis were defined as a T-score below − 1.0, between − 1.0 and − 2.5, and below − 2.5, respectively, in at least one measured region [9].

### Statistical analysis

Binary variables were compared using either the chi-square (χ^2^)  test or Fisher’s exact test as appropriate. Continuous variables were compared using the Wilcoxon rank sum test. Correlation between BMD and mean IMT was evaluated using the Spearman test. The propensity score was calculated for each patient using the nine covariates including age, postmenopausal status, duration of disease, duration of GC use, current dose of GC, statin use, chronic kidney disease, BMD T-score and SLEDAI-2 K, which were considered appropriate for the calculation using the Kolmogorov-Smirnov test (Additional file 1: Table S1). Strata were compared using the Mantel-Haenszel test. All *p* values less than 0.05 were considered significant. All statistical analyses were performed with JMP Pro V.12.0.1 (SAS Institute Inc., Cary, NC, USA).

## Results

**Fig. 1:**
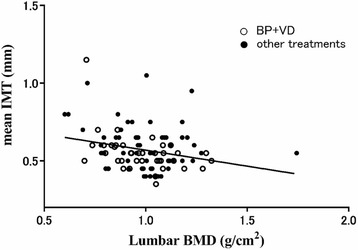
Relationship between lumber spine bone mineral density (BMD) and mean intima-media thickness (IMT) measurements. The Spearman rank correlation coefficient was − 0.25, *p* < 0.01. BP, bisphosphonate; VD, vitamin D agent

A total of 117 patients with SLE were included in this study. As previously reported [10], BMD correlated negatively with mean IMT (Fig. 1). Of the 117 patients, 42 (36%) were receiving BP + VD, 27 (23%) BP alone, 30 (26%) VD alone and 7 (6%) other agents, including denosumab, estrogen, teriparatide and calcium, to treat or prevent osteoporosis. Of the 72 patients receiving BP, 58 (81%) were on alendronate, 7 (10%) risedronate, 6 (8%) minodronate, and 1 (1%) ibandronate.

We first compared the patients’ demographics, BMD measurements, carotid measurements, and medications for SLE between the BP + VD and other treatment groups. Low BMD was more frequent, and carotid plaque was less prevalent in the BP + VD group compared with other treatment groups (Table 1). Among traditional and SLE-related risk factors for atherosclerosis, duration of disease and that of GC use were shorter in the BP + VD group compared with other treatment groups, possibly being associated with the less prevalent carotid plaque. The cumulative dose of GC was strongly correlated with duration of GC use (Spearman correlation coefficient = 0.93). Duration of anti-osteoporotic treatment was not different between the BP + VD and any of the other groups.

**Table 1:** Characteristics of the patients

	BP + VD (n = 42)	Other treatments (n = 75)	*p* value
Demographics			
Female, *n* (%)	35 (83%)	69 (92%)	0.22
Age (years)	40 (31, 49)	43 (35, 54)	0.18
Age at diagnosis (years)	32.5 (21, 43)	28 (19, 40)	0.19
Smoking, *n* (%)	16 (38%)	28 (43%)	0.59
Post menopause, *n* (%)	13/35 (37%)	30/69 (43%)	0.56
BMI (kg/m^2^)	22.3 (20.1, 24.1)	22.1 (19.2, 26.0)	0.56
Duration of disease (years)	5 (0, 13)	12 (5, 24)	< 0.001
SLEDAI-2 K	5 (2, 13)	4 (2, 6)	0.07
History of LN, *n* (%)	20 (48%)	31 (41%)	0.51
APS, *n* (%)	10 (24%)	14 (19%)	0.47
Diabetes mellitus, *n* (%)	9 (21%)	17 (23%)	0.88
Hypertension, *n* (%)	26 (62%)	45 (60%)	0.84
Systolic blood pressure (mmHg)	119 (110, 130)	121 (111, 132)	0.59
Diastolic blood pressure (mmHg)	74 (69, 82)	77 (70, 86)	0.25
Dyslipidemia, *n* (%)	32 (76%)	50(67%)	0.28
Total cholesterol (mg/dL)	194 (160, 226)	199 (172, 220)	0.51
Triglyceride (mg/dL)	130 (95, 185)	147 (94, 209)	0.39
HDL-C (mg/dL)	61 (42, 76)	64 (52, 75)	0.25
LDL-C (mg/dL)	99 (86, 131)	101 (83, 124)	0.87
CKD, *n* (%)	20 (48%)	32 (43%)	0.65
eGFR (mL/min)	79.1 (24.8)	74.1 (25.8)	0.31
Serum creatinine (mg/dL)	0.7 (0.60, 0.88)	0.68 (0.59, 0.87)	0.98
aPL, *n* (%)	17 (40%)	25 (33%)	0.44
History of CVD, *n* (%)	6 (14%)	5 (7%)	0.20
History of bone fractures, *n* (%)	5 (12%)	9 (12%)	1.00
BMD measurements			
BMD (lumbar spine) (g/cm^2^)	0.975 (0.159)	0.990 (0.180)	0.68
BMD (FN) (g/cm^2^)	0.717 (0.151)	0.708 (0.117)	0.61
Low BMD, *n* (%)	25/38 (66%)	23/63 (37%)	0.004
Osteoporosis, *n* (%)	4/38 (12%)	5/63 (12%)	0.73
Carotid measurements			
Carotid plaque, *n* (%)	7 (17%)	33 (44%)	0.002
Mean IMT (mm)	0.56 (0.12)	0.61 (0.17)	0.07
Treatment			
Duration of anti-osteoporotic treatment (months)	47 (3, 72)	47 (26, 65)	0.89
Cumulative dose of GC (g^a^)	37.2 (4.5, 86.2)	74.3 (23.4, 118.3)	0.028
Duration of GC use (months)	80 (2, 166)	164 (47, 276)	0.002
Current dose of GC (mg^a^/day)	16 (9, 30)	7.5 (5, 10)	< 0.001
Concomitant use of IS, *n* (%)	29 (69%)	41 (55%)	0.12
mPSL pulse, *n* (%)	23 (55%)	28 (38%)	0.079
Maximum dose of oral GC (mg^a^/day)	60 (40, 60)	60 (30, 60)	0.38
Antihypertensive agent, *n* (%)	25 (60%)	36 (48%)	0.23
Statin, *n* (%)	24 (57%)	29 (39%)	0.054

Values are number (percent), median (25th, 75th percentiles), or mean (SD)

*BP* bisphosphonate, *VD* vitamin D agent, *SLE* systemic lupus erythematosus, *BMI* body mass index, *SLEDAI-2 K* systemic lupus erythematosus disease activity index 2000, *LN* lupus nephritis, *APS* antiphospholipid syndrome, *HDL-C* high-density lipoprotein cholesterol, *LDL-C* high-density lipoprotein cholesterol, *CKD* chronic kidney disease, *aPL* antiphospholipid antibody, *CVD* cardiovascular disease, *eGFR* estimated glomerular filtration rate, *BMD* bone mineral density, *FN* femoral neck, *IMT* intima-media thickness, *GC* glucocorticoid, *IS* immunosuppressant, *mPSL* methylprednisolone

^a^Prednisolone equivalents

Next, we evaluated factors contributing to the development of carotid plaque by univariate (Table 2) and multivariate (Table 3) analyses. Among factors extracted by the univariate analysis including age, post menopause, duration of disease, history of lupus nephritis, estimated glomerular filtration rate, serum creatinine, history of cardiovascular disease, BMD (of both the lumbar spine and femoral neck), T-scores (of both the lumbar spine and femoral neck), mean IMT, cumulative dose of GC, duration of GC use, current dose of GC, concomitant use of immunosuppressants, antihypertensive agents and BP + VD treatment, only age (OR = 1.57) and BP + VD treatment (OR = 0.24) were shown by multivariate analysis to be promotive and protective factors, respectively. We excluded relevant confounding factors with age, including post menopause, estimated glomerular filtration rate, serum creatinine, mean IMT and antihypertensive agent, as variables in multivariate analysis.

**Table 2:** Comparisons of patients’ characteristics

	Carotid plaque (+) (n = 40)	Carotid plaque (−) (n = 77)	*p* value
Demographics			
Female, *n* (%)	37 (93%)	67 (87%)	0.54
Age (years)	49 (39, 65)	40 (31, 47)	<0.0001
Age at diagnosis (years)	33 (23, 42)	29 (18, 38.5)	0.07
Smoking, *n* (%)	20 (50%)	28 (36%)	0.16
Post menopause, *n* (%)	23/36 (64%)	20/67 (30%)	0.002
BMI (kg/m^2^)	22.6 (19.1, 26.3)	22.3 (19.5, 25.2)	0.87
Duration of disease (years)	17 (6, 25)	7 (1.5, 15)	0.002
SLEDAI-2 K	3.5 (2, 7)	7 (2, 12)	0.07
History of LN, *n* (%)	10 (29%)	37 (49%)	0.04
APS, *n* (%)	11 (28%)	14 (18%)	0.24
Diabetes mellitus, *n* (%)	11 (28%)	15 (20%)	0.35
Hypertension, *n* (%)	29 (73%)	42 (55%)	0.07
Systolic blood pressure (mmHg)	121 (112, 132)	120 (109, 132)	0.47
Diastolic blood pressure (mmHg)	77 (70, 85)	75 (70, 85)	0.77
Dyslipidemia, *n* (%)	31 (78%)	51(66%)	0.29
Total cholesterol (mg/dL)	198 (172, 221)	196 (167, 227)	0.73
Triglyceride (mg/dL)	146 (103, 201)	139 (90, 201)	0.63
HDL-C (mg/dL)	64 (53, 75)	64 (49, 75)	0.69
LDL-C (mg/dL)	104 (85, 126)	100 (81, 125)	0.91
CKD, *n* (%)	15 (38%)	21 (27%)	0.29
eGFR (mL/min)	64.8 (18.9)	81.7 (26.6)	0.0005
Serum creatinine (mg/dL)	0.78 (0.63, 0.90)	0.65 (0.58, 0.84)	0.02
aPL, *n* (%)	19 (48%)	23 (30%)	0.07
History of CVD, *n* (%)	7 (18%)	4 (5%)	0.04
History of bone fractures, *n* (%)	7 (9%)	7 (18%)	0.20
BMD measurements			
BMD (lumbar spine) (g/cm^2^)	0.931 (0.170)	1.008 (0.168)	0.032
T-score (lumbar spine)	−0.75 (1.52)	−0.09 (1.53)	0.048
BMD (FN) (g/cm^2^)	0.652 (0.102)	0.738 (0.134)	0.006
T-score (FN)	−1.26 (0.96)	−0.65 (1.08)	0.02
Low BMD, *n* (%)	19 (56%)	29 (43%)	0.29
Osteoporosis, *n* (%)	6 (18%)	3 (5%)	0.06
Carotid measurements			
Mean IMT (mm)	0.69 (0.21)	0.54 (0.10)	<0.0001
Treatment			
Duration of GC use (months)	210 (46, 296)	98 (18, 191)	0.006
Cumulative dose of GC (g^a^)	88.4 (24.1, 120.8)	48.7 (10.4, 100.6)	0.036
Current dose of GC (mg^a^/day)	6 (5, 10)	10 (7, 28)	0.005
Concomitant use of IS, *n* (%)	18 (45%)	52 (68%)	0.03
mPSL pulse, n (%)	18 (45%)	33 (43%)	0.82
Maximum dose of oral GC (mg^a^/day)	60 (30, 60)	60 (30, 60)	0.41
Antihypertensive agent, *n* (%)	26 (65%)	35 (45%)	0.04
Statin, *n* (%)	20 (50%)	33 (43%)	0.56
BP, *n* (%)	19 (48%)	50 (66%)	0.07
VD, *n* (%)	21 (53%)	55 (71%)	0.07
BP + VD, *n* (%)	7 (18%)	35 (46%)	0.004

Values are number (percent), median (25th, 75th percentiles), or mean (SD)

*BP* bisphosphonate, *VD* vitamin D agent, *SLE* systemic lupus erythematosus, *BMI* body mass index, *SLEDAI-2 K* systemic lupus erythematosus disease activity index 2000, *LN* lupus nephritis, *APS* antiphospholipid syndrome, *HDL-C* high-density lipoprotein cholesterol, *LDL-C* high-density lipoprotein cholesterol, *CKD* chronic kidney disease, *aP*L antiphospholipid antibody, *CVD* cardiovascular disease, *eGFR* estimated glomerular filtration rate, *BMD* bone mineral density, *FN* femoral neck, *IMT* intima-media thickness, *GC* glucocorticoid, *IS* immunosuppressant, *mPSL* methylprednisolone

^a^Prednisolone equivalents

**Table 3:** Multivariate logistic regression analysis

Variables	Odds ratio	95% CI	*p* value
Age (per decade)	1.57	1.04–2.48	0.03
Duration of GC use (months)	1.00	0.997–1.005	0.73
Current dose of GC (mg^a^/day)	0.96	0.90–1.01	0.12
BMD T-score^b^	0.66	0.40–1.04	0.07
BP + VD treatment	0.24	0.07–0.75	0.01

*CI* confidence interval, *GC* glucocorticoid, *BMD* bone mineral density, *BP* bisphosphonate, *VD* vitamin D agent

^a^Prednisolone equivalents

^b^Smaller value of T-score was taken either for the lumber spine or femoral neck measurement

**Fig. 2:**
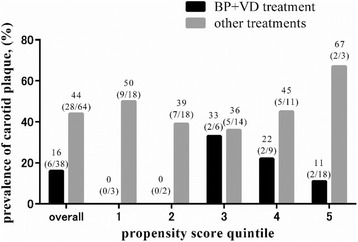
Comparison of the prevalence of carotid plaque in the bisphosphonate (BP) + vitamin D (VD) treatment group and other treatment groups according to propensity score quintile. Values are percent (number of patients with carotid plaque/total number of each group)

To further confirm the protective role of BP + VD against carotid plaque development, we performed an unbiased analysis using propensity scoring. In 15 out of 117 patients, the propensity score could not be estimated due to the unavailability of T-scores. The remaining 102 patients were divided into 5 different strata using the propensity score calculated using age, postmenopausal status, duration of disease, duration of GC use, current dose of GC, statin use, chronic kidney disease, T-score and the SLEDAI-2 K (Additional file 1: Table S1) and ranged from 0.01 to 0.97. The borderlines of these five strata were 0.12, 0.28, 0.41 and 0.62. Stratum 1 included the patients with the lowest propensity score while stratum 5 included patients with the highest. Carotid plaque was less prevalent in the BP + VD group compared with other treatment groups in all 5 strata (Fig. 2). The difference was considered significant using the Mantel-Haenszel test (*p* = 0.015).

## Discussion

To our knowledge, this is the first study to demonstrate that the combination of BP + VD has a potential to retard atherosclerosis in patients with SLE. Our data also showed that BMD correlated negatively with IMT in patients with SLE, as previously reported [10]. Atherosclerosis in SLE could be affected not only by traditional risk factors but also by SLE-related risk factors, such as duration of disease, duration of glucocorticoid use, disease activity, antiphospholipid antibodies, and renal manifestations [1]. Statins have been shown to prevent atherosclerosis in the general population by lowering low-density lipoprotein cholesterol levels and by multiple off-target effects, such as anti-inflammation and proliferation [11]. A prospective randomized trial in 200 patients with SLE, however, failed to show the anti-atherosclerotic effect of the statins, indicating that statins alone may not be sufficient to prevent atherosclerosis in SLE [12].

Bisphosphonates are expected to inhibit arterial plaque development and calcification through several mechanisms [13]. Nitrogen-containing bisphosphonates and statins inhibit the mevalonate pathway through interaction with farnesyl pyrophosphate synthase to prevent post-translational modification of proteins, resulting in decreased levels of inflammatory cytokines and matrix metalloproteinases [14, 15]. Bisphosphonates also decrease a variety of mature vascular cells, which migrate into the vessel walls and injure vascular endothelial cells [16, 17]. A clinical trial with elderly osteoporotic women, however, did not show an anti-atherosclerotic effect of ibandronate [18]. A systematic review and meta-analysis recently performed by Kranenburg et al. [19] demonstrated the effect of bisphosphonates on reduction in arterial wall calcification. Of note, treatment with statins in combination with bisphosphonates was more effective in terms of reducing atherosclerotic plaque compared with either monotherapy in patients with hypercholesterolemia [20], indicating the additive anti-atherosclerotic effect of bisphosphonates with statin therapy.

Vitamin D deficiency is emerging as a novel risk factor for CVD [5]. Vitamin D has been shown to protect endothelial cells from oxidative stress and subsequent apoptosis. Low levels of 25-dehydroxyvitamin D were associated with increased cardiovascular events, whereas the effect of vitamin D supplementation to prevent CVD is now under investigation [21]. Although Vitamin D has been shown to improve SLE disease activity [22], a prospective randomized trial with 114 postmenopausal women did not demonstrate an anti-atherosclerotic effect of vitamin D [23]. Conversely, Robinson et al. [24] demonstrated that vitamin D status may determine the effect of statin on carotid IMT in juvenile SLE.

Based on these findings, we hypothesized that the combined treatment using bisphosphonates and vitamin D may be more effective compared with bisphosphonate or vitamin D alone in the prevention of atherosclerosis. Furthermore, combination BP + VD is commonly indicated in the treatment of SLE, since most patients require adequate anti-osteoporotic treatment during long-term glucocorticoid use. Subsequently, several issues arise concerning anti-resorptive therapy, including the duration of BP use due to concern about atypical femoral fractures, and the timing of switching to denosumab, teriparatide or other agents due to inadequate efficacy.

This study has some limitations. First, this was a cross-sectional single-center retrospective study in Japanese patients only. As with all retrospective study design, anti-osteoporotic drugs were selected according to the physicians’ decision, leading to potential bias in grouping. Although propensity scoring was used to minimize the effect of other confounding factors, further prospective multicenter studies and randomized controlled trials are needed to confirm the results of this analysis. Second, serological data on biomarkers associated with osteoporosis, such as vitamin D status, inflammatory cytokine levels, parathyroid hormone and homocysteine, were not available, due to the absence of measurements. The daily calcium intake also could not be calculated due to the absence of data on supplementation and eating habits. Additional laboratory testing may better elucidate the interplay of specific biomarkers in both osteoporosis and atherosclerosis. Third, at least theoretically, the anti-atherosclerotic effect of a triple combination of statins, bisphosphonates and vitamin D may be more promising but was not well-evaluated herein due to the relatively small sample size. The prevalence of statin use was not different in patients with and without carotid plaques (Table 2).

## Conclusions

This study provides some preliminary evidence to support the use of combination therapy with bisphosphonates and vitamin D to prevent atherosclerosis in patients with SLE. Both bisphosphonates and vitamin D supplementation are already recommended for osteoporosis, and their combination may prevent the development and progression of atherosclerotic plaques better than either agent alone. Further large-scale prospective studies are warranted to confirm the results of this analysis.

## Additional file

Additional file 1: **Table S1.** *P* values for each variable in each stratum for comparison between the BP + VD treatment and the other treatment groups after propensity score adjustment. (DOCX 19 kb)

## Abbreviations

ACR: American College of Rheumatology
BMD: Bone mineral density
BP: Bisphosphonate
CVD: Cardiovascular disease
GC: Glucocorticoids
IMT: Intima-media thickness
SD: Standard deviations
SLE: Systemic lupus erythematosus
SLEDAI-2 K: Systemic lupus erythematosus disease active index 2000
VD: Vitamin D
