# Supplementary material for: Effect of combined treatment with bisphosphonate and vitamin D on atherosclerosis in patients with systemic lupus erythematosus: a propensity score-based analysis
Source: Arthritis Res Ther. 2018 Apr 17;20:72. doi: 10.1186/s13075-018-1589-9 (PMC5905171; doi:10.1186/s13075-018-1589-9)
Supplement: Supplementary file 1 — Table S1. P values for each variable in each stratum for comparison between the BP + VD treatment and the other treatment groups after propensity score adjustment. (DOCX 19 kb) [file 13075_2018_1589_MOESM1_ESM.docx]

Table S1. P values of each variable for each stratum between BP+VD treatment and the other treatment groups after propensity score adjustment.

|  | Stratum | | | | |  |
| --- | --- | --- | --- | --- | --- | --- |
| **Variables** | 1 | 2 | 3 | 4 | 5 | |
| Female | 1.00 | 1.00 | 1.00 | 1.00 | 1.00 | |
| Age (years) | 0.54 | 0.51 | 1.00 | 1.00 | 0.99 | |
| Post menopause | 1.00 | 0.62 | 1.00 | 0.50 | 0.43 | |
| Duration of disease (year) | 0.94 | 0.76 | 1.00 | 0.24 | 0.06 | |
| SLEDAI-2K | 0.94 | 0.95 | 0.24 | 0.95 | 0.83 | |
| History of LN | 1.00 | 1.00 | 0.60 | 0.18 | 1.00 | |
| Hypertension | 0.59 | 1.00 | 0.61 | 1.00 | 1.00 | |
| CKD | 0.53 | 0.48 | 1.00 | 0.37 | 0.55 | |
| eGFR (mL/min) | 0.54 | 0.76 | 0.50 | 0.86 | 0.69 | |
| Serum creatinine (mg/dL) | 0.41 | 0.76 | 0.66 | 0.68 | 0.83 | |
| History of CVD | 1.00 | 1.00 | 0.06 | 0.57 | 1.00 | |
| T-score of BMD* | 0.94 | 1.00 | 0.88 | 0.75 | 0.20 | |
| Low BMD | 0.53 | 1.00 | 0.30 | 0.37 | 0.55 | |
| Mean IMT (mm) | 0.69 | 0.76 | 0.66 | 0.99 | 0.99 | |
| Cumulative dose of GC (g**) | 0.98 | 0.58 | 0.79 | 0.89 | 0.07 | |
| Duration of GC use (month) | 0.94 | 0.64 | 0.99 | 0.49 | 0.09 | |
| Current dose of GC (mg**/day) | 0.61 | 0.44 | 0.94 | 0.09 | 0.99 | |
| Concomitant use of IS | 1.00 | 1.00 | 1.00 | 0.89 | 0.49 | |
| Antihypertensive agent | 0.59 | 1.00 | 0.32 | 0.79 | 1.00 | |
| Statin | 1.00 | 1.00 | 1.00 | 1.00 | 0.55 | |

Binary variables were compared using either χ^2^ test or Fischer’s exact test, continuous variables compared using Kolmogorov-Smirnov test of strata balance after propensity score adjustment.

*Smaller value of T-score was taken either lumber or femoral neck.

**Prednisolone equivalents

BP: bisphosphonate, VD: vitamin D agent, BMI: body mass index, SLE: systemic lupus erythematosus, SLEDAI-2K: SLE disease activity index 2000, LN: lupus nephritis, CKD: chronic kidney disease, eGFR: estimated glomerular filtration rate, CVD: cardiovascular disease, BMD: bone mineral density, F.N: femoral neck, IMT: intima-media thickness, GC: glucocorticoid, IS: immunosuppressant
